# Supplementary material for: Super-resolution time-resolved imaging using computational sensor fusion
Source: Sci Rep. 2021 Jan 18;11:1689. doi: 10.1038/s41598-021-81159-x (PMC7813875; doi:10.1038/s41598-021-81159-x)
Supplement: Supplementary file 1 — Supplementary Information [file 41598_2021_81159_MOESM1_ESM.pdf]

Supplementary Information: Super-Resolution Time-Resolved Imaging using Computational Sensor Fusion

C. Callenberg[1], A. Lyons[2], D. den Brok[1], A. Fatima[2], A. Turpin[3], V. Zickus[2], L. Machesky[4], J. Whitelaw[4], D. Faccio[2], M.B. Hullin[1]
[1] *Institute of Computer Science, University of Bonn, Germany*
[2] *School of Physics & Astronomy, University of Glasgow, G12 8QQ Glasgow, United Kingdom*
[3] *School of Computing Science, University of Glasgow, G12 8LT Glasgow, United Kingdom and*
[4] *Cancer Research UK, Beatson Institute, Glasgow, United Kingdom*

Light Transport and Image Formation

A light-in-flight image is a three-dimensional data cube with two spatial and one temporal dimension. It can be understood as an image where each spatial pixel consists not of a single value like in a conventional intensity image, but of a temporal histogram which contains information about how much light the pixel receives at a given time t after the emission from a light source. Following the notation of O'Toole et al. [1], the flux of photons Φ for each pixel at time t is then

$$\Phi(t) = (s * l)(t) + a(t) \tag{1}$$

where s is the scene response defined by the geometry and reflectance of the scene, l is the temporal distribution of the illumination pulse, and a is the ambient light present in the scene. This distribution is sampled using the SPAD sensor which produces a signal for each detected photon that is then time-stamped by the respective timing electronics. All detected photon events are then sorted into a temporal histogram. For each SPAD pixel, a histogram is measured:

$$n(t) = \eta_{\text{SPAD}} (\Phi * j)(t) + \gamma_{\text{SPAD}} \tag{2}$$

where η_{SPAD} is the photon detection probability of the SPAD, j is the jitter accounting for uncertainties in the time-stamping, and γ_{SPAD} denotes dark counts of the sensor. Temporal discretisation is determined by the width of each histogram bin. The whole light-in-flight image is a spatial grid of these temporal histograms and therefore has the form of a three-dimensional data cube $i(x, y, t) = n_{x,y}(t)$.

In addition to the SPAD sensor, we utilize a CCD sensor that sees the same image as the SPAD sensor, but naturally has no time resolution. It can thus only measure the integrated signal

$$p = \eta_{\text{CCD}} \int_{\Delta T} \Phi(t)\, \mathrm{d}t + \gamma_{\text{CCD}} \tag{3}$$

that sums up all light intensity measured during an exposure time ΔT by the sensor with quantum efficiency η_{CCD}, including dark counts γ_{CCD}. The whole two-dimensional CCD image is a grid of pixel values $c(x, y) = p_{x,y}$.

In the following mathematical considerations, all images are vectorized, e.g. the vector c contains all pixels of a CCD image in a linear sequence, a vector d analogously contains all entries of the light-in-flight data cube.

In our set-up, both sensors share the same objective lens via a beam splitter. Due to this fixed imaging system, only one single initial alignment calibration is necessary to ensure that the time-integrated SPAD-image matches the CCD-image. It can easily be performed using a calibration target like a printed pattern and then choosing the appropriate crop of the CCD sensor that matches the integrated SPAD image.

Forward Model and Reconstruction of High-Resolution Light-in-Flight Images

Our goal is to fuse a low spatial resolution SPAD image of size $m \times n \times \tau$ (a data cube with $m \times n$ spatial pixels and τ time bins) with an intensity image of dimension $M \times N$ in order to recover a high resolution data cube of the size $M \times N \times \tau$. To achieve this, the light transport from the scene to the SPAD sensor is described by a matrix A of size $m \cdot n \times M \cdot N$, which models all effects that the signal undergoes in the spatial domain on its way from the high

resolution state as it is measured by the CCD camera, to the low resolution state as measured by the SPAD array. In principle, this transport matrix could be gauged experimentally by illuminating the scene with suitable patterns and probing the mapping from CCD pixels to SPAD pixels. This process is difficult to conduct in practice, as the illumination would have to be modified in such a way that it is capable of illuminating only certain exact pixels in the CCD image. To circumvent this tedious calibration step, we instead develop the matrix as a forward model based on the known (linear) effects on the signal, which can then also be used to produce simulated SPAD measurements from a high-resolution ground truth. The matrix A in our model is given as the product of three matrices:

$$A = P \cdot S \cdot B \tag{4}$$

each representing a distinct step in the transport.

As described in the main paper, the SPAD sensor is moved slightly out of focus in order to acquire information from all scene points despite the low fill factor of the sensor. Instead of calibrating for the point spread function of the system, the blur that is accounted for in the matrix B is approximated as a 2D Gaussian. In synthetic experiments with different blur kernels, a Gaussian kernel yielded the best reconstruction results, even when the simulated measurements had been produced using other possible shapes like a disk kernel (which would correspond to the isolated defocus blur as it constitutes a convolution with the aperture shape) instead. B is thus the convolution matrix that, when multiplied with a vectorized image, yields the convolution of said image with a Gaussian kernel.

The matrix S acts as a mask, corresponding to the distribution of the active pixel area on the sensor, selecting the part of the incident light that is actually measured by the SPAD pixels. In practice, S is a diagonal matrix containing only zeros and ones - depending on whether a high resolution image pixel falls onto passive or active SPAD sensor area.

The matrix P performs the downsizing of the resulting image from $M \times N$ to $m \times n$ pixels by summing up corresponding patches of pixels (for $k \times k$ downscaling, P would add up patches of size $k \times k$ to obtain a SPAD pixel value).

The matrix A acts only on the spatial dimensions of the light-in-flight image and is thus applied on each temporal bin of the light-in-flight data cube:

$$r = A_\tau \cdot i_{\mathrm{HR}}, \tag{5}$$

where r is the vectorized raw SPAD measurement, i_{HR} is the vectorized high-resolution transient image and $A_\tau \in \mathbb{R}^{mn\tau \times MN\tau}$ applies A to all time bins of i_{HR}. It is obtained as the Kronecker product of an identity matrix of size $\tau \times \tau$ and the matrix A:

$$A_\tau = \mathbb{1}_\tau \otimes A \tag{6}$$

More details on the matrices used in the model can be found in the section below.

In the case of noisy SPAD data (real measurement or simulations including noise), the data cube is first denoised using total variation in all three dimensions. The contrast of the CCD image is adjusted such that a potential offset of the pixel values is removed in order to eliminate sensor specific noise. Its intensity is furthermore scaled to match the integrated intensity of the SPAD measurement, so that the total intensity integrated over all pixels and time bins is the same for the CCD and the SPAD measurement. This is necessary due to different quantum efficiencies and exposure times, as well as different measurement units of the sensors and corresponds to a scaling of factor

$$f = \frac{\eta_{\mathrm{SPAD}} \cdot \Delta T_{\mathrm{SPAD}}^{\mathrm{eff}}}{e \cdot \eta_{\mathrm{CCD}} \cdot \Delta T_{\mathrm{CCD}}} \tag{7}$$

under the assumption of temporally constant ambient light, with an effective exposure time $\Delta T_{\mathrm{SPAD}}^{\mathrm{eff}}$ of the SPAD sensor and a conversion factor e between photoelectrons and digital pixel intensity values. These individual quantities constituting f need not be known; instead we use

$$f = \frac{\sum_{x,y,t}^{m,n,\tau} d(x,y,t)}{\sum_{x,y}^{M,N} c(x,y)}, \tag{8}$$

treating intensity values in the SPAD and CCD images as effectively dimensionless quantities and units as implicit.

Forward Model Details

In order to fuse a low spatial resolution SPAD measurement of size $m \times n \times \tau$ (a data cube with $m \times n$ spatial pixels and τ time bins) with an intensity image of dimension $M \times N$, we first model the light transport from the scene to the SPAD sensor. It is described by a matrix A of size $m \cdot n \times M \cdot N$ that is the product of three matrices, as given in Eq.4, where P, S and B each represent a distinct step in the transport and are described in more detail in the following. All matrices are given for a toy example of a 4×4 SPAD array with $\tau = 16$ time bins and a CCD image of resolution 16×16.

Since our SPAD sensor is moved out of focus, we model the resulting blur by a 2D Gaussian distribution. The matrix B then looks as shown in Fig. 1. When multiplied with a vectorized image of resolution $M \times N$ (represented as a column vector of length $M \cdot N$), it yields a blurred image of the same resolution (plus additional padding due to the blur).

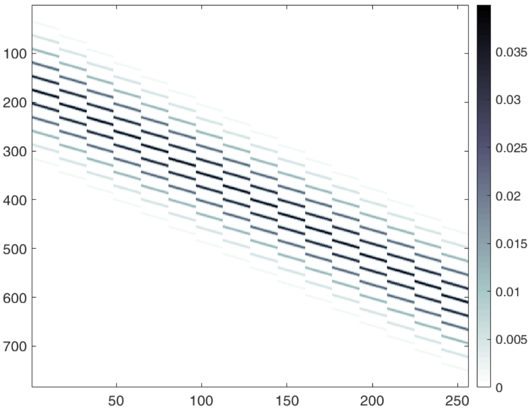

FIG. 1: Matrix B.

The SPAD sensor has a low fill factor. We therefore include in our model the fact that only 2% of the light actually reaches the active pixel area and neglect the rest of the light. A mask as shown on the left side of Fig. 2 is used to model the distribution of active area on the sensor area. It is reshaped into a matrix that can be multiplied with the blurred vectorized image, as can be seen on the right side of Fig. 2.

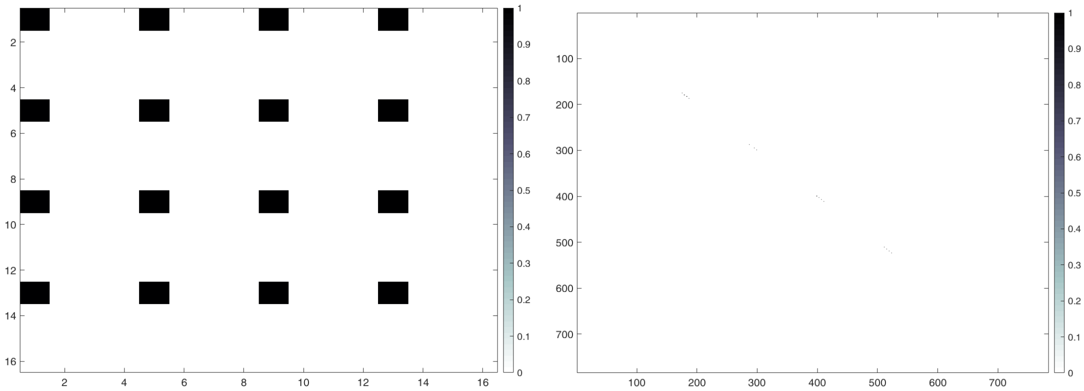

FIG. 2: Left: Mask of the active pixel areas on the whole sensor area. Right: Matrix S - due to the dimensions of S, the active areas are only barely visible as black dots on the diagonal.

Lastly, the matrix P performs the downscaling of the vectorized $M \times N$ image (plus padding from the blur) to the resolution $m \times n$ by summing up respective image areas (in this case patches of 4×4 pixels - see Fig. 3).

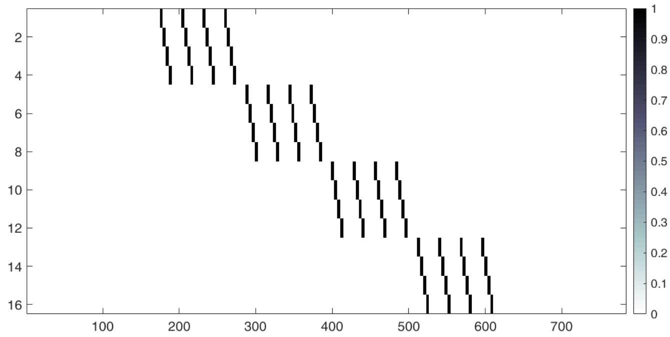

FIG. 3: Matrix P.

The matrix A which is the product of the matrices P, S, and B, then looks as shown in Fig. 4.

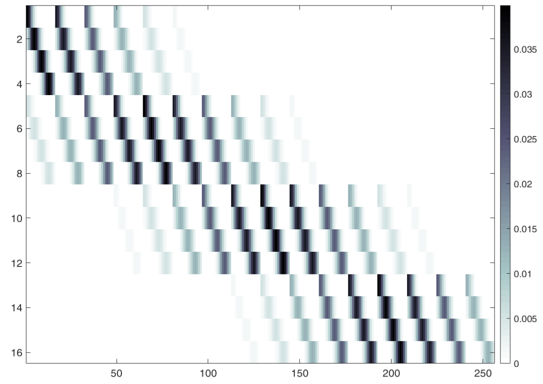

FIG. 4: Matrix A.

Since this matrix acts only on a single image and not on a full SPAD data cube, we construct a matrix A_τ as the Kronecker product of an identity matrix of size $\tau \times \tau$ and the matrix A:

$$A_\tau = \mathbb{1}_\tau \otimes A$$

When multiplied with a vectorized high resolution data cube x_{HR} (size $M \cdot N \cdot \tau \times 1$), it yields a low resolution SPAD measurement r (size $m \cdot n \cdot \tau \times 1$), including blur and mask, by applying A to each time frame of the data cube:

$$d = A_\tau \cdot i_{\text{HR}}.$$

A_τ is of dimension $m \cdot n \cdot \tau \times M \cdot N \cdot \tau$ as depcited in Fig. 5.

Using this forward model, a high resolution data cube can be reconstructed from a low resolution SPAD measurement and a high resolution CCD image as described in the paper.

Numerical Simulations

In order to simulate the fusion of a CCD sensor image and a corresponding SPAD measurement, we created artificial light-in-flight images of three-dimensional scenes by using a time-of-flight renderer[1] that raytraces the scene, stores the time each ray has travelled from the light source to the camera and sorts them into a histogram for each camera pixel. The result is a three-dimensional data cube of the light-in-flight image. An integration over the temporal dimension of the high-resolution ground-truth rendering serves as the simulated CCD image (see e.g. Fig. 7). Simulated SPAD

---

[1] Further information on the used renderer can be found in [2].

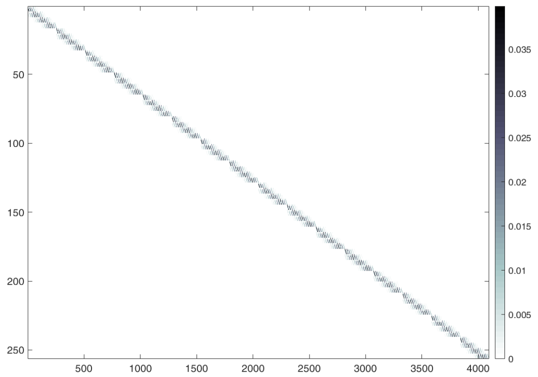

FIG. 5: Matrix A_τ.

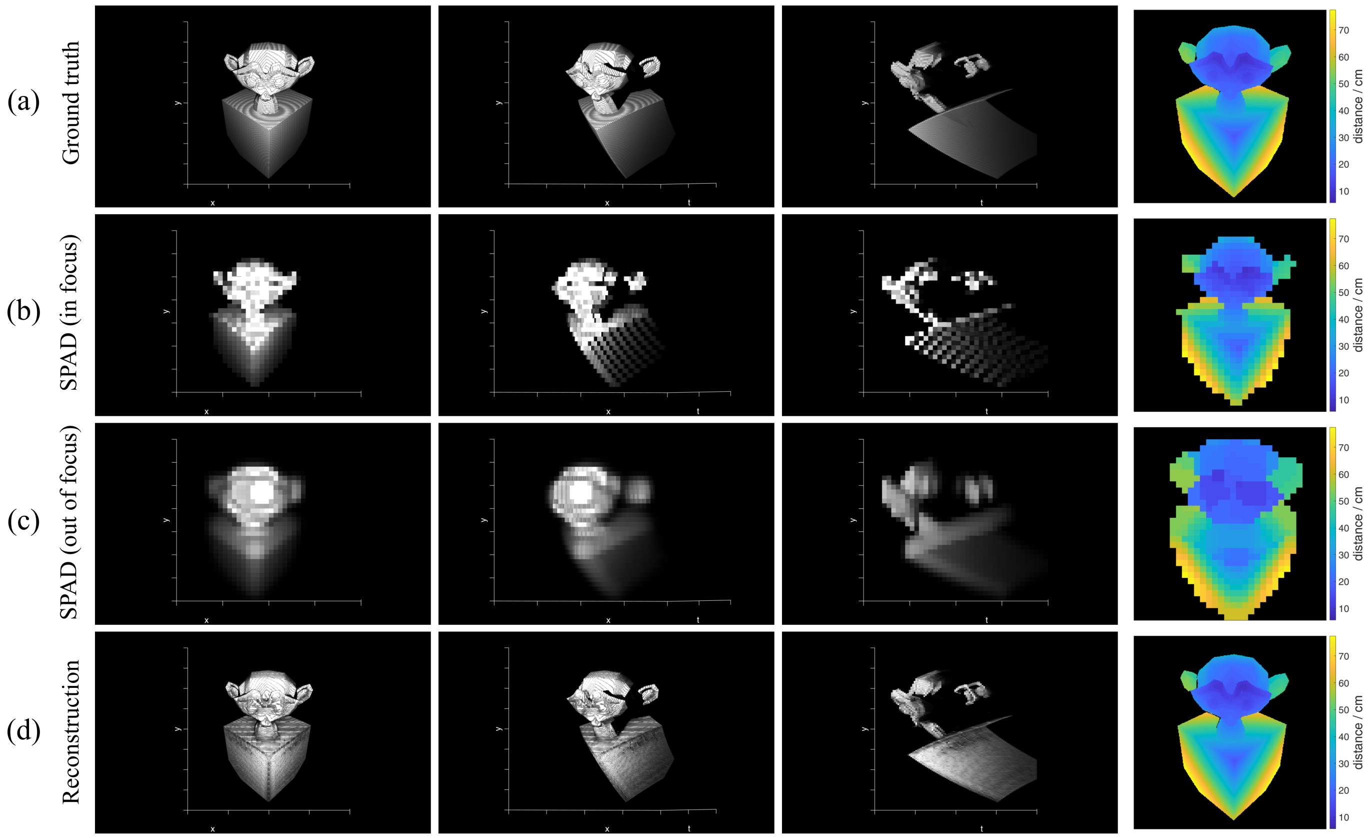

FIG. 6: (a) High resolution ground truth simulated light-in-flight image. (b) The same scene in simulated SPAD sensor view when the image is in focus. (c) Simulated SPAD view when the image is out of focus. (d) Reconstruction from the out-of-focus measurements and a high-resolution time-integrated image of the scene. Columns 1-3 show a volume rendering of the scene from different perspectives, column 4 shows a depth image of the scene obtained by using the time bin with the highest photon count as the depth information per pixel.

measurements are created from this high-resolution data cube using the forward model described earlier. Figure 6 shows the light-in-flight data cube as volume renderings seen from different angles and, for additional visualisation, as depth maps. These depth maps are created in a naive way, using the time bin with the highest photon count as depth information, and are meant to provide additional visualisation for scenes with negligible amounts of multiply scattered light. They should not be considered comprehensive visualisations of the reconstruction results as they are not created using state-of-the-art methods and always constitute a reduction of the data to two dimensions. This holds for all depth maps depicted in this paper. In Fig. 6, the ground truth data cube is shown in row (a) and the corresponding simulated SPAD measurements created using the forward model in row (c). The scene is visibly blurred

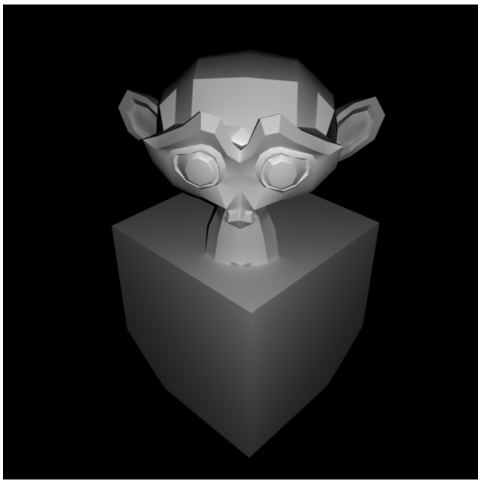

FIG. 7: Simulated CCD image of the artificial three-dimensional scene.

in the spatial dimension – a standard variation of 6 high-resolution pixel widths was used for the Gaussian kernel. Row (b) shows what the SPAD measurement would look like without any blur: due to the low fill factor of the SPAD sensor, information is lost and holes appear in the image in the temporal dimension due to spatially sparse sampling.

Using only the simulated measurement from (c) and the simulated high-resolution CCD image, the scene can be reconstructed as seen in row (d) according to Eq. 3 of the main text. Suitable regularization parameters α, β and γ were found experimentally by performing a parameter sweep over several orders of magnitude for each parameter. The total variation prior was found to be unnecessary for this scene, as it contains mostly direct reflections, thus δ was set to zero. $\alpha = 1$, $\beta = 10^{-7}$ and $\gamma = 10^{-4}$ were found to yield the best and most stable reconstruction results and were used for all reconstructions of this simulated scene throughout this paper.

Figure 8 shows reconstructions of the same scene in different spatial resolutions with and without noise. The width of the Gaussian blur used in the simulation was adjusted for each resolution, from $\sigma = 1.5$ CCD pixel widths in the 96×96 case to $\sigma = 6$ CCD pixel widths in the 384×384 reconstruction. The noise is modeled as a combination of Poissonian and Gaussian noise in order to account for shot noise as well as other effects such as thermal or readout noise. In order to model the simulated measurements as closely to the real measurements as possible, the pixel count range of the artificial data was adjusted to typical values measured with the set-up described in the main paper before applying the Poisson noise (roughly $5 \cdot 10^6$ total photon counts, which corresponds to ∼100 photon counts per pixel and time bin - meaning per 'voxel' in the data cube - on average). Additionally, areas in the measured data cube (after background subtraction) that do not contain any signal (only noise) were analysed and the pixel values were found to follow a Gaussian distribution (due to background subtraction having already been applied here, noise that is actually of Poissonian nature with high mean values is treated as a Gaussian distribution with lower mean). The parameters of this distribution were fitted to the data and used for the artificial Gaussian noise that was added to the simulated data. As a result, signal-independent Gaussian noise with $\mu = 5.6$ and $\sigma = 6.1$ was added to the simulated data, in addition to the Poisson noise.

As measures of the reconstruction performance, the peak signal-to-noise ratio (PSNR) as well as the relative error $\delta_{\mathrm{rel}} = {}^{||i_{\mathrm{reco}} - i_{\mathrm{truth}}||_2}/_{||i_{\mathrm{truth}}||_2}$ are stated for each resolution. While in the noise-free case the quality of the reconstruction is approximately constant, the addition of noise decreases the reconstruction performance both numerically and perceptually. The degradation also increases with higher spatial upsampling factors. However, even for a spatial resolution of $M \times N = 384 \times 384$, which corresponds to a factor of 12 in both spatial dimensions of the SPAD measurement, a meaningful reconstruction could be achieved even in the presence of noise. In principle, there is no fundamental limit to the resolution that could be gained in this way. The reconstructions would, however, degrade as the upsampling ratio is increased. This could be alleviated with a more precise knowledge of the optical system to ensure the forward model matches well the experimental conditions. One must also consider computational time for larger dataset – one route to reducing the processing time is to sub-divide the scene into smaller spatial "patches" in which case the computational time would scale linearly with pixel count.

An additional simulated scene featuring a diffuse table, three diffuse walls forming a corner, and a specular mirror located behind the table, is shown on the left side of Fig. 9. Due to inter-reflections between the objects, a light-in-flight image of this scene has a complex temporal distribution that can not be reduced to single light bounces on

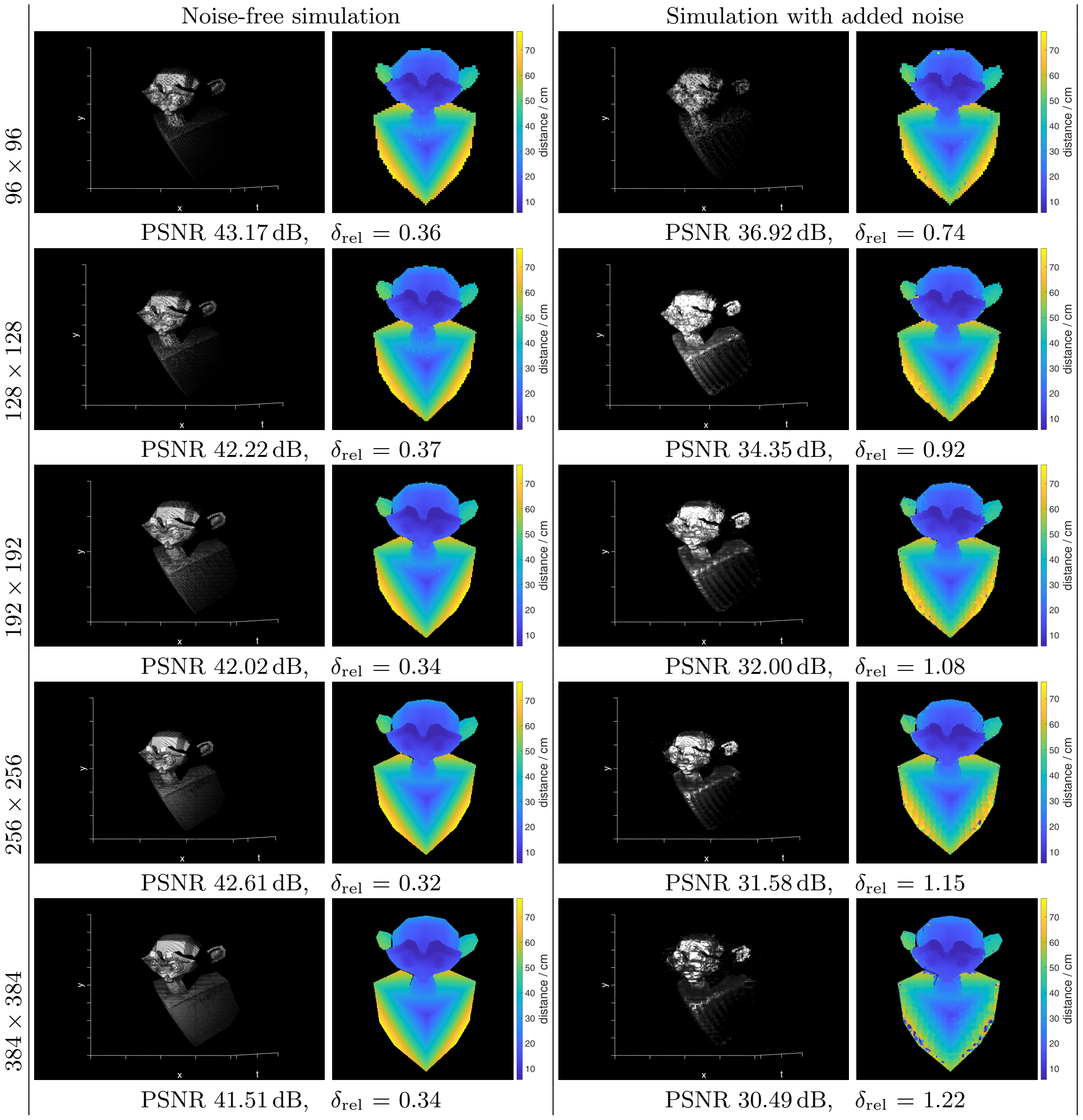

FIG. 8: Reconstructed simulated scene in different spatial resolutions (96×96 to 384×384) with 95 time bins without (left column) and with (right column) added noise. For each reconstruction, the deviation from the ground truth is given as the peak signal-to-noise ratio (PSNR) and the relative error $\delta_{\mathrm{rel}} = {}^{||i_{\mathrm{reco}} - i_{\mathrm{truth}}||_2}/_{||i_{\mathrm{truth}}||_2}$. Both refer to the whole three-dimensional light-in-flight image, not the depth image.

each surface. On the right side of Fig. 9, the temporal intensity profile of a spatial pixel of the light-in-flight image (marked by a red square in the left image) is shown: After an initial peak of light that undergoes a single reflection on its path from the light source to the camera, additional, less intense light is detected by the same pixel, due to interreflections of the surrounding walls.

Figure 10 shows five frames from the simulated light-in-flight image at different points in time, the first row depicting the ground truth, the second the 32×32 pixels measurement that has been created from it. The third row shows the reconstruction of a 256×256 light in flight image from the 32×32 SPAD measurement and the simulated CCD image (as shown in Fig. 9, left). It uses the same parameters as above, except $\delta = 10^{-7}$, therefore employing a total variation prior. The last row shows how the reconstruction quality degrades when Gaussian and Poisson noise is added (using the same scaling and parameters as above). This and Fig. 9 (right) show that the reconstruction matches the ground truth well, with some artefacts in the late time-frames that contain only multiply reflected light, especially with added noise. The time frame at $t = 1.430$ ns (column four) shows how spatial detail is resolved in the reconstruction that is not visible in the simulated measurement due to the low resolution.

Since the intensity level of the multiply reflected light is very low (only about 5% of the intensity of the direct

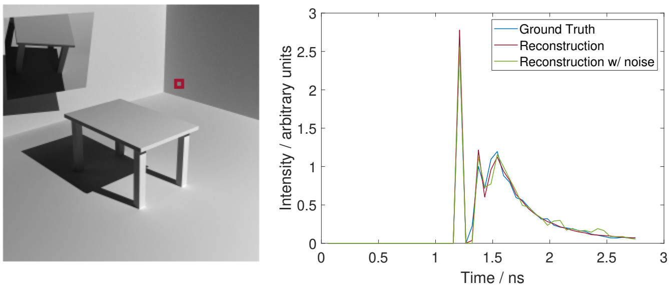

FIG. 9: Simulated scene featuring a diffuse table and walls, as well as a specular mirror behind the table. The red square marks the spatial pixel whose temporal light intensity profile is plotted on the right. After a primary (direct) reflection, additional light that has scattered off the surrounding walls is reflected towards the camera from this same scene point.

reflections, as can be seen from the color legends in Fig. 10), its reconstruction is affected strongly by additional noise. If necessary, lower noise levels can be achieved in experiment by averaging multiple measurements of the same scene, which would extend the acquisition time accordingly.

The supplemental material of the paper contains video renderings of the light-in-flight image and its reconstructions.

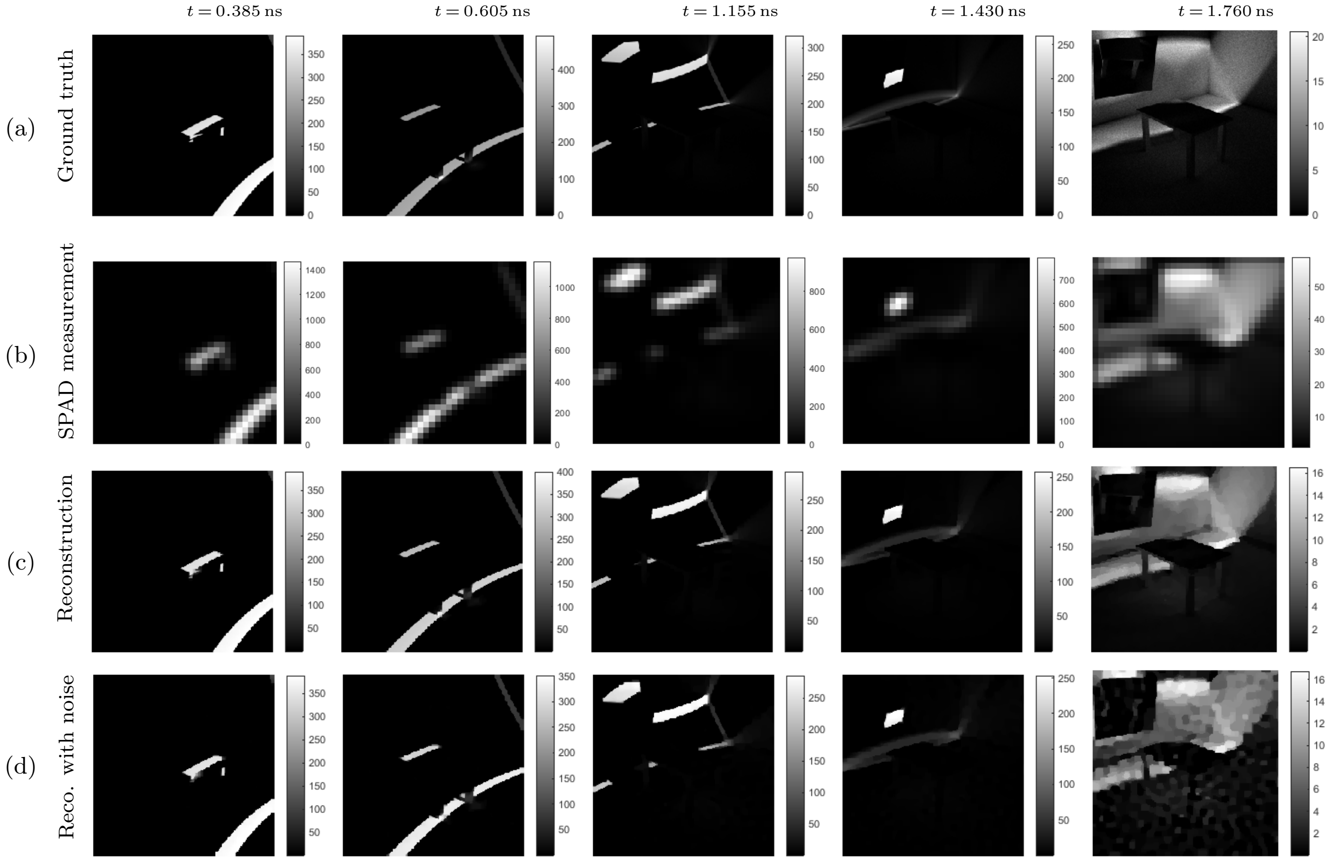

FIG. 10: Five frames from the light-in-flight image of the scene shown on the left side of Fig. 9 at different points in time (see column headings) illustrating multiple light bounces in the scene. Due to severe differences in brightness between direct and higher order reflections, each frame's brightness has been adjusted to the respective depicted intensity range (see color legends). (a) Ground truth simulation. (b) Simulated SPAD measurement of size 32×32 (without added noise). (c) Reconstruction to 256×256 without added noise. (d) Reconstruction to 256×256 with added noise.

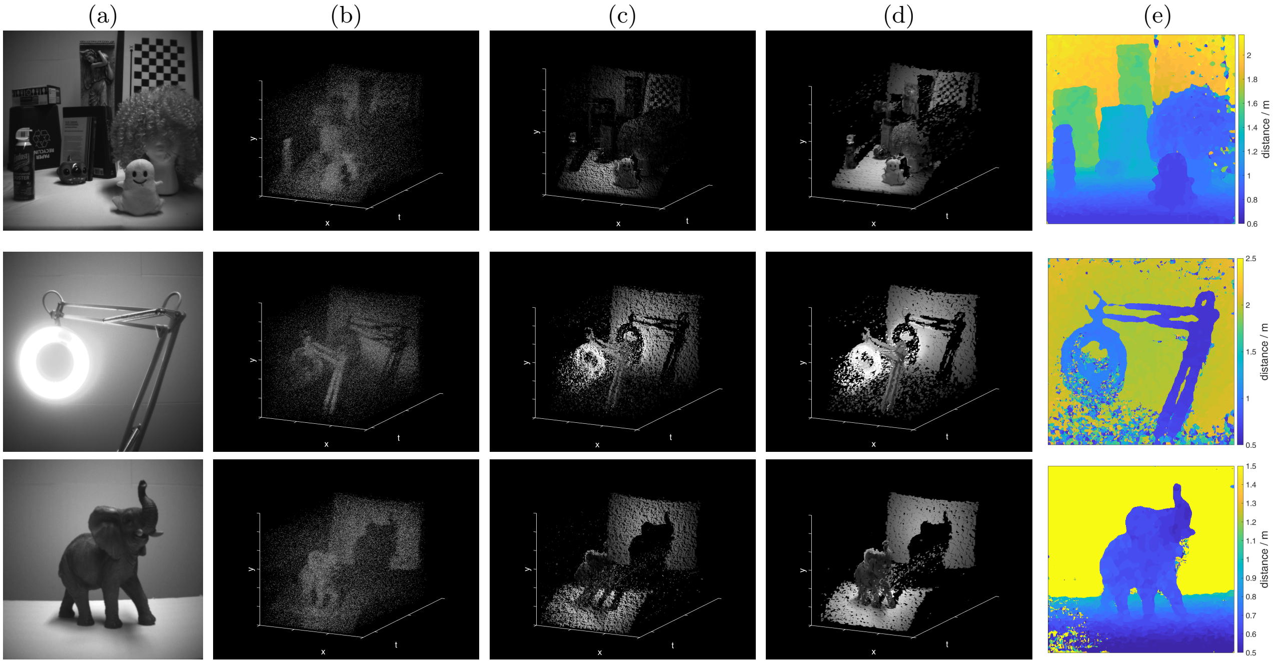

FIG. 11: (a) CCD image and (b) SPAD image from [3] [4]. (c) Reconstruction using our method in full temporal resolution. (d) Reconstruction using our method after temporally rebinning the SPAD data sixfold. (e) Simple depth images created from the rebinned reconstruction.

Image Retrieval Runtimes

Data set	Time bins τ	Run time / mins
Golfball	25	11.6
Waterglass	44	78.3
Basketball	34	37.8
Letters	51	126.7
Steps	32	31.3
Simulation	38	19.0

TABLE I: Reconstruction runtimes for data sets depicted in Fig. 2 of the main paper, as well as the simulated data. All reconstructions have a spatial resolution of 96×96 pixels.

Upsampling Results on Other Data Sets

In order to evaluate and compare our method further, we used it to reconstruct high resolution light-in-flight images from measured and simulated data provided by Lindell et al. [3, 4]. Each real mesurement data set consists of a SPAD measurement in 256×256 pixels spatial resolution and a time bin width of 26 ps, as well as a 1024×1024 pixel CCD image of the same scene. The data was captured with the SPAD sensor in focus, so in order to simulate experimental conditions as in our set-up and thus make the data compatible to our reconstruction, we blurred the SPAD measurements spatially with a two-dimensional Gaussian and then downscaled it to a spatial resolution of 64×64 pixels. We then used our method to upsample this 'simulated out-of-focus measurement' data to 512×512 and 1024×1024 for the full time resolution and a temporally rebinned version of the data cube [2], respectively.

[2] According to [3] the FWHM of their acquisition system is ∼440 ps, which corresponds to approximately 17 time bins. We therefore perform a temporal rebinning of factor 6 for faster reconstrucion times and smoother results.

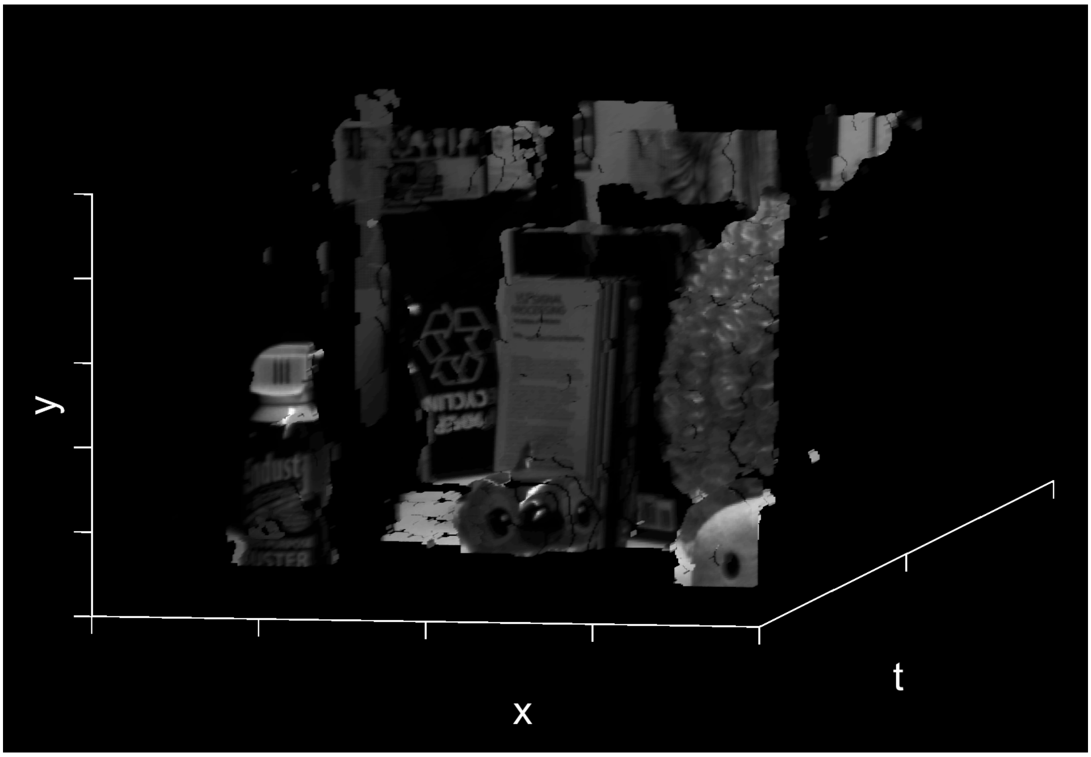

FIG. 12: Detail of the first scene shown in Fig. 11 from a slightly different angle. It shows how high resolution detail from the CCD camera has been fused into the reconstructed data-cube, including fine intensity details like letters and symbols on objects in the scene.

Figure 11 shows these results for three different datasets. Especially the results depicted in the first row demonstrate how high resolution detail, including intensity information, from the CCD image has been transferred into the reconstructed light-in-flight image (see detail in Fig. 12). For additional illustration and to provide a certain degree of comparability to the upsampling results in [3], (naive) depth images are shown in the last column.

Figure 13 shows details of reconstructions, depicted as (naive) depth maps, from a simulated dataset [4] with very low signal-to-noise ratio (on average 2 signal photons and 50 background photons per pixel). As with the real measurements, to make the data compatible to our method, we first blurred the (now synthetic) SPAD image spatially and downsampled it by a factor of 3. We then upsampled it back to original size using our reconstruction method. For comparison, results from Lindell et al. for the same details can be found in [3] in Figure 4. Despite being noisier than theirs, our depth maps demonstrate that our model is capable of dealing with very low signal levels and produce meaningful depth maps even though no sophisticated method to extract the depth map from the reconstructed light-in-flight image is used.

Reconstructions of both captured and simulated data from [4] show a certain level of "patchiness" in the depth maps, which can also be observed in a video view of the reconstructed data cubes (see supplemental material). Since this behaviour is also visible in reconstructions on simulations with high signal-to-noise ratio, and not present in our own captured data, we suspect that it is caused by the artificial blur being applied to the SPAD measurement in a resolution of only 256×256 pixels (as this is the maximum resolution available). The original scene signal at this point has already undergone sampling by a SPAD device, including all uncertainties and losses that come with it (low fill factor, spatial quantization). Using this data to create the 64×64 measurement that serves as input for our method, and then upsampling it to 1024×1024, is basically an attempt to reconstruct more details than the undefocused SPAD data contains, justifying a lack in quality (i.e. in the form of 'patchiness') in the results. With the optical blur during the acquisition, before the sampling step, we specifically address this problem by spreading scene information into multiple pixels. Better results would therefore be expected from data that had been acquired with a set-up like ours, as it would include more scene information in the SPAD measurement than we can account for with the given measurement data sets.

In comparison to the work by Lindell et al. [3], our method is capable of upsampling whole light-in-flight images

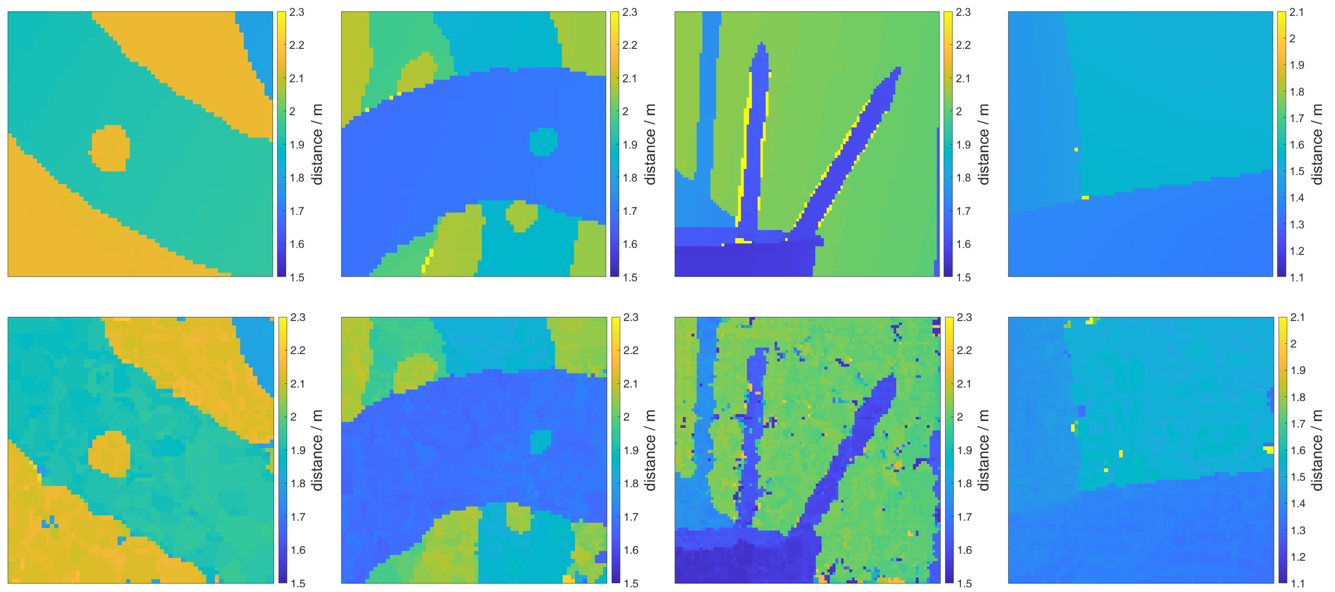

FIG. 13: Depth maps of details from a scene simulated with low signal-to-noise ratio of only 2 signal photons and 50 background photons per pixel. Top: ground truth. Bottom: Results using our reconstruction.

including mutliply reflected light. Instead of a neural network, it employs convex optimization and an appropriate image formation model. It is still possible to reconstruct depth maps from the reconstructed scenes in cases where this mapping makes sense (mostly direct reflections). Our method is not limited to, but allows usage of a low resolution two-dimensional SPAD array that does not require scanning of the scene, like the set-up used by [3]. As we downsample their 256×256 SPAD data by a factor of 4×4 after blurring it, we demonstrate upsampling by a factor of up to 16×16 on these external data sets (from 64 to 1024 pixels edge length).

---

[1] Matthew O'Toole, Felix Heide, David B Lindell, Kai Zang, Steven Diamond, and Gordon Wetzstein. Reconstructing transient images from single-photon sensors. In *Proceedings of the IEEE Conference on Computer Vision and Pattern Recognition*, pages 1539–1547, 2017.

[2] Jonathan Klein, Martin Laurenzis, Dominik L. Michels, and Matthias B. Hullin. A quantitative platform for non-line-of-sight imaging problems. In *British Machine Vision Conference 2018, BMVC 2018, Northumbria University, Newcastle, UK, September 3-6, 2018*, page 104, 2018.

[3] David B Lindell, Matthew O'Toole, and Gordon Wetzstein. Single-photon 3d imaging with deep sensor fusion. *ACM Transactions on Graphics (TOG)*, 37(4):113, 2018.

[4] Stanford Computational Imaging Lab. Single-photon 3d imaging with deep sensor fusion.
